# Peer Review of Scientific Studies: Problems and Potential Solutions

**DOI:** 10.7759/cureus.95357

**Published:** 2025-10-24

**Authors:** Sunanda Gupta, Aruddha Sarkar

**Affiliations:** 1 Community and Family Medicine, Central Armed Police Forces Institute of Medical Sciences (AIIMS-CAPFIMS), New Delhi, IND; 2 Arthroscopy and Sports Injury, Kalinga Hospital Limited, Bhubaneshwar, IND

**Keywords:** artificial intelligence, bias, health research, peer review, publication, research review, review, reviewer, review system, scientific article

## Abstract

Peer review has long stood as the principal safeguard for scientific credibility, yet much of its authority rests on tradition rather than empirical proof of efficacy. In recent years, persistent vulnerabilities, ranging from bias and inconsistency to opaque procedures and protracted delays, have eroded trust in the peer review system. Rising submission volumes, mounting commercial influences, and dwindling reviewer engagement have amplified the strain. Problems span structural and individual levels: an overburdened reviewer base, lack of standardized practices, unclear decision-making, slow turnaround times, and limited diversity in evaluation panels, together with personal pitfalls such as unconscious bias, conflicts of interest, poor accountability, inadequate training, and breaches of confidentiality, are present in the spectrum of issues. This editorial explores practical and ethical reforms to strengthen the process, including elevating reviewing to a recognized profession, introducing meaningful incentives, incorporating artificial intelligence judiciously, embracing transparent yet protective models, expanding reviewer diversity, and streamlining editorial workflows.

## Editorial

Revered for decades as the gatekeeper of scientific truth, peer review ironically operates on trust more than on tested evidence. What began as a noble effort to ensure quality, credibility, and integrity has, over time, revealed deep cracks viz. bias, inconsistency, delay, and opacity. Despite its flaws, no alternative has yet matched its role in research. However, the explosion of global submissions, rising commercial pressures, and limited reviewer motivation now threaten its very sustainability. Empirical evidence indicates that lapses in peer review have contributed to the publication of low-quality or even retracted research, ultimately diminishing scientific credibility and public trust. Reforming peer review is not merely a procedural requirement; it is a moral imperative to preserve the integrity of science.

Systemic challenges

There are a number of challenges, including: (i) Overburdened reviewers: A limited number of experts are repeatedly called upon to evaluate a growing volume of submissions. This excessive workload leads to reviewer fatigue, rushed assessments, and avoidable delays in the publication process. (ii) Reviewer scarcity: Editors often struggle to find qualified and willing reviewers, particularly for niche or interdisciplinary topics. The shortage results in over-reliance on a small cohort of reviewers, compromising timeliness. (iii) Lack of standardization: There are no universally accepted criteria or formalized training for conducting peer reviews. Consequently, the rigor of evaluations varies significantly across journals. (iv) Opaque processes: The mechanisms by which reviewers are chosen and editorial decisions are made remain largely undisclosed. This lack of transparency undermines trust in the fairness and consistency of peer review outcomes. (iv) Lengthy timelines: Multiple review rounds, editorial revisions, and administrative bottlenecks can stretch over many months. Such delays impede the timely dissemination of research findings and are often beyond the control of authors. (v) Lack of diversity: Reviewer panels are frequently dominated by researchers from high-income countries, established institutions, or specific disciplines. This narrow representation limits the inclusivity and global relevance of scientific evaluation. (vi) Incentive gap: Reviewers receive little recognition or tangible reward for their critical scholarly contributions. The absence of incentives diminishes motivation and contributes to the growing reluctance to accept review invitations.

Reviewer level concerns

There are some concerns related to reviewers, including: (i) Bias: Although manuscripts are anonymized during peer review, subtle cues such as institutional and departmental affiliation, author gender, or geographical references often reveal the authors’ identities. These implicit signals can inadvertently introduce bias into the reviewers’ evaluation process. (ii) Conflict of interest: Although conflicts of interest are routinely declared, the extent to which these disclosures are verified remains uncertain and largely dependent on the reviewers’ personal integrity. (iii) Lack of accountability: Anonymous peer review, while designed to promote candour, can sometimes encourage careless, dismissive, or even biased remarks. The apparent absence of identifiable responsibility may reduce the incentive for reviewers to maintain professionalism and courtesy. (iv) Untrained reviewers: Many reviewers receive little formal guidance on ethical, objective, and constructive reviewing practices. As a result, feedback quality varies widely, and critical methodological or ethical flaws may be easily overlooked. (v) Plagiarism or idea theft: A small but concerning fraction of reviewers misuse the privileged access to unpublished data or novel ideas. Such breaches of confidentiality not only harm authors but also damage trust in the scientific community. (vi) Inconsistent feedback: Authors frequently receive conflicting or incompatible reviewer recommendations, reflecting the subjective nature of the process. This inconsistency can delay publication decisions and frustrate researchers seeking clear guidance for improvement. (vii) Misuse of artificial intelligence (AI): An emerging challenge is the misuse of AI tools for generating superficial or unverified peer reviews, which risks compromising the quality and accountability of scholarly evaluation. Such practices not only undermine the integrity of the peer-review process but also ruin trust in the authenticity of critical appraisal. As the volume of submissions grows and AI enters academic workflows, the need to reform peer review has never been more urgent. Several practical and ethically sound strategies can enhance both the credibility and sustainability of the system.

Potential reforms

Recognize Reviewers as Professionals

For decades, peer review has been perceived and treated as invisible, unpaid labour for the sake of science. This has created reviewer fatigue. Recent arguments strongly suggest that reviewing should evolve into a recognized academic profession, with trained and accredited referees who are compensated for their time, expertise, and effort [1]. This shift would bring accountability, attract qualified reviewers, and reduce the current over-reliance on goodwill. Professionalization does not negate voluntary reviewing but provides a parallel track for complex, high-stakes manuscripts.

Incentivize and Sustain Engagement

Even beyond payment, recognition is essential. A sustainable system would link reviewer contributions to measurable credit through ORCID-linked review records, reviewer indexes, or promotion criteria [2]. Journals and institutions should provide tangible rewards such as continuing education credits, charge waivers, or priority handling of submissions of reviewers. These incentives would acknowledge peer review as a scholarly duty at par with teaching, research, and other duties. Several journals have begun recognizing peer reviewers through tangible incentives, a progressive step that underscores the value of rigorous and timely peer review.

Integrate AI Responsibly

AI offers unprecedented opportunities to improve efficiency: detecting plagiarism, identifying image manipulation, checking adherence to reporting standards, and triaging low-quality submissions [3]. Yet, as Bergstrom and Bak-Coleman note, peer review is ultimately a human activity of science [4]. AI should assist, not replace, human judgment. Safeguards must be in place to prevent misuse. Editorial team oversight is critical to ensure transparency, fairness, and data security.

Enhance Transparency Without Sacrificing Safety

Traditional peer review is opaque, eroding trust among authors and readers. Experiments with open peer review, where review reports are published alongside articles, can demonstrate increased accountability [5]. However, anonymity remains important to protect junior or vulnerable reviewers. A hybrid model, in which reviews are made public, but identities are revealed only if the reviewer consents, balances transparency with safety.

Build Diversity and Equity into Reviewer Pools

Peer review too often reflects inequities in science. Reviewers are disproportionately drawn from high-income countries, senior positions, and only established institutions. Broader, global reviewer databases are needed to ensure diverse perspectives and reduce bias [2]. Formal mentorship, pairing senior reviewers with early-career professionals, can expand capacity while training the next generation.

Streamline and Standardize Review Processes

Excessive delays frustrate authors. Simple structural reforms, such as reviewer templates, structured checklists, and cross-journal portability of reviews, can reduce redundancy and improve consistency [2]. Sharing reviews across journals, with author consent, avoids duplication of labour. Templates focusing on methodology, reporting, and ethics discourage irrelevant or hostile comments and help editors make decisions faster.

As a human endeavour, peer review will never be flawless, but it can be improved to become faster and fairer. Professionalizing reviewing, incentivizing true engagement, integrating AI responsibly, balancing transparency with safety, ensuring diversity, and streamlining processes are suggested as feasible steps. As Emanuele and Minoretti argue, the peer review crisis demands radical reform [1]. To sustain its role as the bedrock of scientific publishing, peer review must be re-imagined not as an unpaid obligation but as a recognized, accountable, and evolving profession.

## References

[1] Emanuele E, Minoretti P (2025). The peer review crisis demands radical reform: why reviewers should become paid professional referees. Cureus.

[2] Aczel B, Barwich AS, Diekman AB (2025). The present and future of peer review: Ideas, interventions, and evidence. Proc Natl Acad Sci U S A.

[3] Perlis RH, Christakis DA, Bressler NM, Öngür D, Kendall-Taylor J, Flanagin A, Bibbins-Domingo K (2025). Artificial intelligence in peer review. JAMA.

[4] Bergstrom CT, Bak-Coleman J (2025). AI, peer review and the human activity of science. Nature.

[5] Routledge D, Pariente N (2025). On improving the sustainability of peer review. PLoS Biol.

